# Hybrid optical and thermal simulation and experimental evaluation of linear fresnel receivers with single and dual non-vacuum tubes

**DOI:** 10.1038/s41598-026-52808-w

**Published:** 2026-05-21

**Authors:** Salman Brbhan, Ahmed Al Mers, Yousra Filali Baba, Zoltán Szamosi, Baibhaw Kumar, Katalin Voith

**Affiliations:** 1 Institute of Energy Engineering and Chemical Machinery, University of Miskolc, Miskolc, 3515 Hungary; 2 ISI laboratory, Abdelamalek Essaadi University, National School of Applied Sciences, Tetouan, Morocco; 3 Thermal and Energy Research Team (ERTE), Ecole Nationale Supérieure d’Arts et Métiers of Rabat (ENSAM-R), Mohammed V University in Rabat, Rabat, Morocco

**Keywords:** Linear Fresnel receiver, Single- and dual-tube design, Monte Carlo Ray-Tracing, CFD, Experimental validation, Performance, Energy science and technology, Engineering, Mathematics and computing, Physics

## Abstract

Non-vacuum linear Fresnel receivers with secondary reflectors are prone to considerable convective and radiative heat losses, while experimental evaluation remains costly and time-consuming. This study develops a hybrid numerical methodology that combines Monte Carlo Ray-Tracing (MCRT) for optical analysis and Computational Fluid Dynamics (CFD) for thermal modelling, validated against experimental measurements on a single-tube receiver operated up to approximately 320 °C. The comparison shows excellent agreement, with deviations below 5% in global heat loss predictions, supporting the ability of the model to reproduce the overall heat balance of the reference configuration. The validated approach is then applied to assess and compare single- and dual-absorber tube designs integrated within a CPC-type cavity. Both designs achieve similar optical efficiencies (around 65%), while the dual-tube configuration is predicted to yield slightly lower overall heat loss coefficients (4.6–10.3 vs. 4.9–10.8 W/m²·K) over absorber tube temperatures ranging from 173 to 473 °C. Under the modelling assumptions and boundary conditions considered, this trend is associated with the greater spacing between the smaller-diameter tubes and the glass cover, which may mitigate radiative and convective heat losses to the ambient. Overall, The results suggest that the dual-tube configuration can offer a modest thermal advantage without significantly affecting optical efficiency. The proposed hybrid modelling approach provides a reliable and cost-effective alternative to extensive experimental testing for the comparative assessment and optimization of non-vacuum Fresnel receivers.

## Introduction

In the global pursuit of sustainable energy, concentrated solar power (CSP) and concentrated solar thermal (CST) technologies stand at the forefront of large-scale renewable heat and power generation^1^. Among them, the Linear Fresnel Concentrator (LFC) has rapidly gained attention as a promising alternative to conventional Parabolic Trough Collectors (PTCs), showcasing good balance between thermal performance and cost-effectiveness^2^. Its inherent simplicity, flat or slightly curved mirrors focusing sunlight onto a stationary receiver, translates into a technology that is both robust and adaptable across diverse climatic and industrial contexts^3^. Yet, despite its potential, the industrial deployment of LFC systems remains limited, and our understanding of their full performance potential is still evolving. Unlike PTCs, LFC systems are particularly suited for medium-temperature applications (100–250 °C), offering an attractive solution for process heat generation, including enhanced oil recovery (EOR), water desalination, agro-food processing, and chemical manufacturing^4,5^.

Functionally, the technology relies on a precise linear focusing mechanism, where direct normal irradiance (DNI) is reflected by an array of reflective mirrors toward a fixed receiver positioned above the mirror field^6,7^. Within this configuration, the absorber tube captures concentrated radiation and converts it into usable heat, a deceptively simple process that conceals complex optical and thermal interactions still being actively explored^8^.

Recent studies have advanced our understanding of LFC receivers. MATLAB-based optical models predict optical efficiencies and guide optimal receiver geometries for maximum solar flux capture^9^. Experimental validation and simulation studies emphasize the influence of temporal and geographic factors on solar power prediction, improving receiver validation strategies^10^. CFD-based 3D steady-state analyses in ANSYS Fluent reveal how circumferential heat flux distributions vary with tube geometry, directly informing absorber design^11^, while integrated thermal and electrical simulations using GREENIUS software allow performance evaluation of full-scale CSP plants^12^. Empirical correlations coupled with CFD provide insight into combined conduction, convection, and radiation phenomena under dynamic conditions^13^.

Studies exploring surface or cavity enhancements demonstrate higher optical performances than conventional PTC, and comparative analyses show LCOE improvement in the non-evacuated case^14^. Secondary optics such as Compound Parabolic (CPC) and Elliptic (CEC) Concentrators fine-tune acceptance angles and flux uniformity, revealing trade-offs between optical gain and angular range^15^. Mirror frameworks coupling optical and thermohydraulic optimization have further shown significant energy utilization gains through controlled reflector defocusing^16^.

One of the standout advantages of LFC systems over traditional PTCs is their fixed-receiver design, which provides unmatched flexibility for technical refinement and economic optimization. Such flexibility enables the integration of non-vacuum receivers, composed of a transparent glass cover, an absorber tube, and a secondary reflector shaped as a compound parabolic concentrator. Beyond this configuration, alternative designs including trapezoidal cavities^17^ with single or multiple absorber tubes have been explored. Unlike the vacuum tube receivers commonly employed in PTC systems^18^, non-vacuum designs may offer potential cost advantages and provide opportunities for further performance optimization^19^. Indeed, the design of evacuated-tube receivers is generally determined by the manufacturer and follows a standard configuration. In addition, Key geometrical characteristics including the absorber tube diameter, glass envelope diameter, and the cylindrical geometry of the tube, are therefore predefined and offer limited flexibility. In contrast, non-evacuated receivers, such as CPC- cavity receivers, allow for a wider range of design options that can be tailored to the overall solar field layout. This flexibility provides engineers and designers with multiple opportunities for techno-economic optimization, including adjustments to geometric configuration, dimensions, and material selection.

Despite considerable progress in modelling and experimental studies of solar receivers, a pronounced gap persists in the systematic characterization of non-vacuum receivers. These receivers, while simpler and more cost-effective to manufacture and maintain than their vacuum-based counterparts, present challenges due to their susceptibility to convective and radiative heat losses. A rigorous and integrated evaluation framework is therefore essential to accurately quantify their performance and guide practical implementation.

Yet, despite these advancements, a crucial question remains open: How the numerical modelling can be effectively employed to accurately reproduce the real thermal behaviour and heat balances of non-vacuum linear Fresnel receivers, while minimizing the reliance on costly and time-consuming experimental procedures. Addressing this challenge represents one of the methodology focus presented in this article. Indeed, to address this critical need, the present study introduces a comprehensive methodology that gathers optical and detailed thermal analyses with experimental validation of moderate complexity, ensuring practical feasibility, specifically targeting receivers with non-vacuum tubes within a CPC-type cavity. Compared to the conventional single-tube configuration, the developed approach is used to demonstrate how the two-tube configuration can improve overall thermal performance, thereby providing practical insights to enhance operational efficiency.

On this basis, the present study develops a modelling framework to capture the full behavior of non-vacuum CPC receivers. The methodology combines Monte Carlo Ray-Tracing (MCRT)^20^ for high-fidelity optical simulation with CFD^21^ to accurately model optical-thermal phenomena, offering a truly comprehensive assessment of performance. A distinctive feature of this approach is that it bypasses the need to explicitly simulate heat transfer fluid circulation, significantly simplifying the analysis without compromising precision. Experimental validation further ensures the model’s reliability in reproducing energy balances, allowing confident prediction of receiver performance under realistic steady-state conditions. By simultaneously accounting for optical interactions and detailed heat transfer, the framework enables a deep understanding of receiver behavior. Important to note that the geometrical parameters of the CPC cavity were optimized within the Sirocco Project^22^, ensuring a design that maximizes efficiency. Finally, the validated methodology is applied to a comparative study of single- versus dual-tube configurations positioned within the same cavity, providing actionable insights into how two-tube design affect thermal and optical performance, and ultimately guiding the design of more efficient, cost-effective, and deployable non-vacuum solar receivers.

## System description

**Fig. 1:**
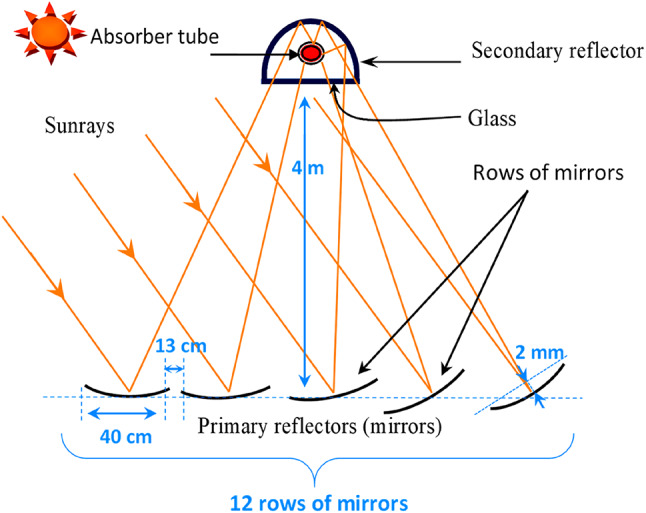
Sketch of Linear Fresnel Concentrator (LFC) using slightly curved mirrors and no-vacuum receiver with secondary reflector. Modified Ref^19^.

The Linear Fresnel Reflector (LFR) system primarily consists of an array of flat or slightly curved mirror rows mounted on a single-axis tracking structure oriented along the North–South direction (Fig. 1). This configuration allows the mirrors to continuously follow the apparent motion of the sun throughout the day, concentrating the reflected solar radiation onto a fixed linear receiver. Figure 1 shows also a key geometrical characteristics of the linear Fresnel concentrator studied including the bending of the curved mirrors, receiver high, gap between rows and mirrors width. These geometric parameters were obtained through a detailed optimization study based on the PSO algorithm^22^. The optimization process was designed to identify the geometric configurations that maximize the concentrator’s optical efficiency. Additional information on the procedure is available in reference^22^.

**Fig. 2:**
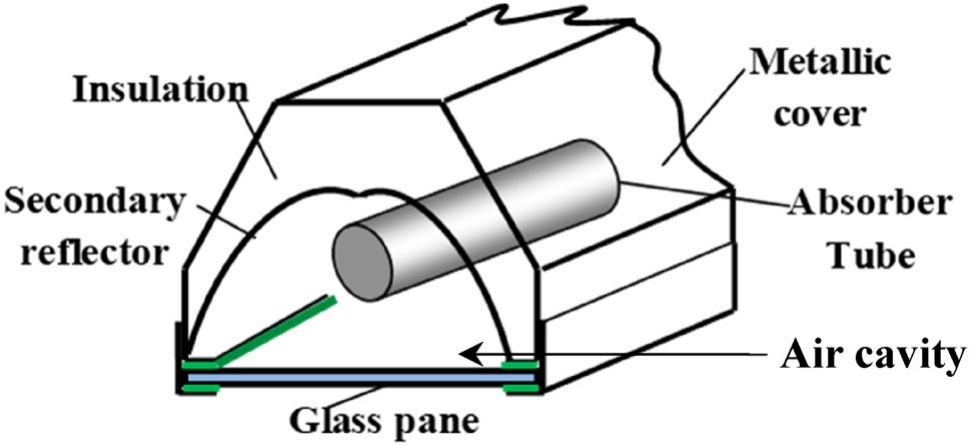
Sketch of no-vacuum receiver with compound parabolic secondary reflector. Modified Ref^19^.

The receiver positioned at a specific height above the mirror field, it includes an absorber tube housed inside an air cavity, CPC secondary reflector, transparent cover made of glass, and back insulation (Fig. 2).

## Methodology

The workflow combines numerical modeling and experimental validation, ensuring both the predictive accuracy and the physical representativeness of the results. The adopted methodology is structured into four interdependent stages.

The study starts by defining the geometric, optical, and thermal parameters of the investigated receiver configurations, including among several parameters the absorber tube diameter, CPC geometry, material optical properties (reflectivity, transmissivity, absorptivity) and solar data in term of DNI (Direct Normal Irradiance).

The optical analysis (Sect. 3.1) employs a MCRT approach to determine the solar heat fluxes received by each receiver cavity surfaces and optical efficiency. These results are later used as a volumetric heat source in the heat transfer model.

**Fig. 3:**
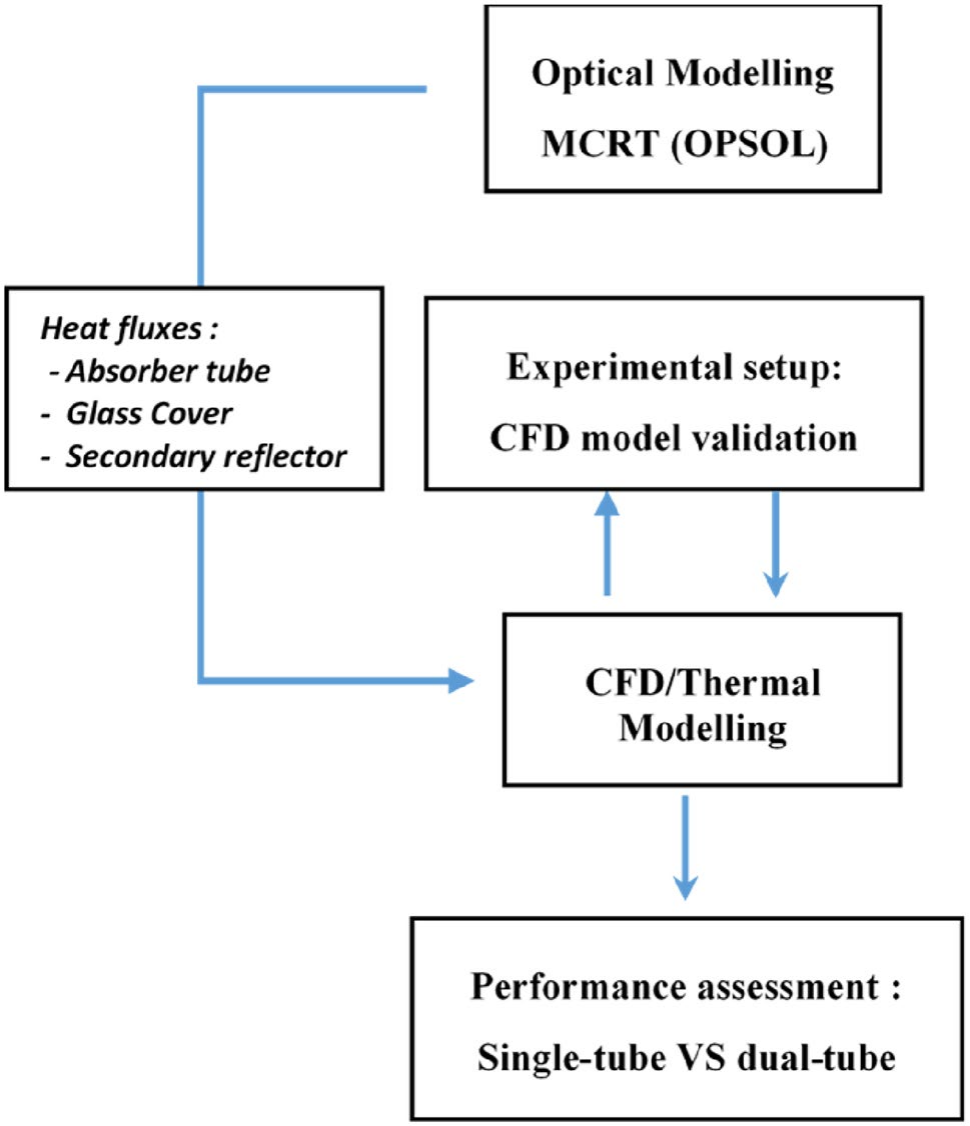
Methodological Framework for Comparative Performance Assessment of Single and Dual tube Non-Vacuum Receivers.

This is followed by the thermal analysis (Sect. 3.2), where CFD simulations assess heat transfer characteristics and temperature fields within the receiver. The accuracy of the results are then verified through experimental validation (Sect. 3.3) using a 2 m real-scale prototype receiver to measure and correlate heat losses under controlled conditions. The comparison between the experimental and simulated results was primarily intended to demonstrate that the CFD approach is capable to reproduce the heat balance with acceptable accuracy, yielding a validated model for assessing and comparing single and dual tube designs. Figure 3 recapitulates the methodological framework designed to thoroughly analyse the performances of single and dual tubes integrated within CPC-type receiver. The following subsections further develop the optical and thermal modeling as well as the experimental stage to offer a comprehensive description of the approach employed.

### Optical analysis (MCRT model)

**Fig. 4:**
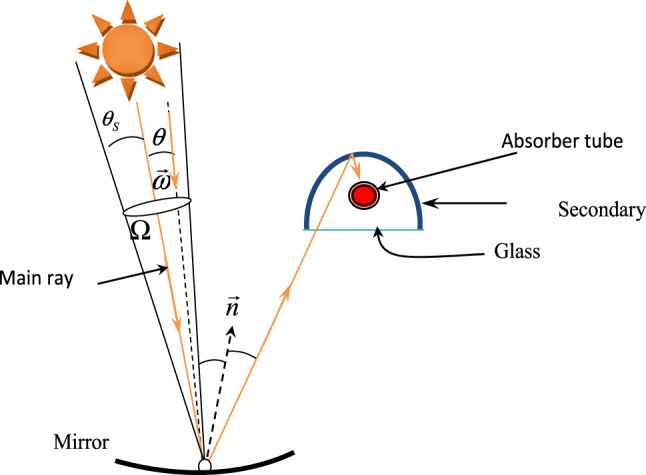
Modelling of the interaction between sun rays and the optical system (for a single mirror).

By using the OPSOL code developed and validated previously^20^, the 3D optical modeling of the LFC is based on a Monte Carlo method combined with a ray-tracing algorithm. Based on this approach, the heat flux density generated on the surface of the absorber tube can be expressed as a double integral defined over the solar disk and over the reflecting surface of the mirrors (Fig. 4)^20^.The total incident heat flux on the absorber tube surface is given by:

1 $$\Phi = \int_C {\;\int_H {\;\left[ {I\;\left( \theta \right)\,{{\vec \omega }_{in}}\;.\;\vec n} \right]\;} } \;\tau \,\prod\limits_1^K {{\rho _k}\,\;} d{S_H}\,d\Omega$$

Where C represents the solar cone defined by the solid angle, including all incident directions $\:\overrightarrow{{\omega\:}_{in}}$ from the sun. H denotes the total reflective surface of the primary reflectors (mirrors). This integral only considers rays that reach the absorber tube after passing through the glass and/or after a series of reflections on the secondary reflector. The term $\:\tau\:\prod\:_{1}^{K}{\rho\:}_{K}$ represents the attenuation factor ($\:\prod\:_{1}^{K}\:$represents the product from 1 to K) of the solar flux intensity $\:I\left(\theta\:\right)$, accounting for transmission effects through the glass cover (with transmission coefficient τ) and for multiple reflections occurring on the mirrors and the secondary reflector. K denotes the total number of reflections, and ρ_k_ is the corresponding reflection coefficient. The solar flux intensity $\:I\left(\theta\:\right)$ is evaluated as a function of the incidence angle$\:\:\theta\:$. This approach allows the angular distribution of the incoming solar flux to be accurately represented based on measured solar irradiance values (DNI) in (W/m^2^).

The use of the Monte Carlo method consists in transforming the deterministic problem represented by the integral (1) into a probabilistic one. The formulation of a Monte Carlo algorithm provides a formal mathematical framework for the analysis^7^.

**Fig. 5:**
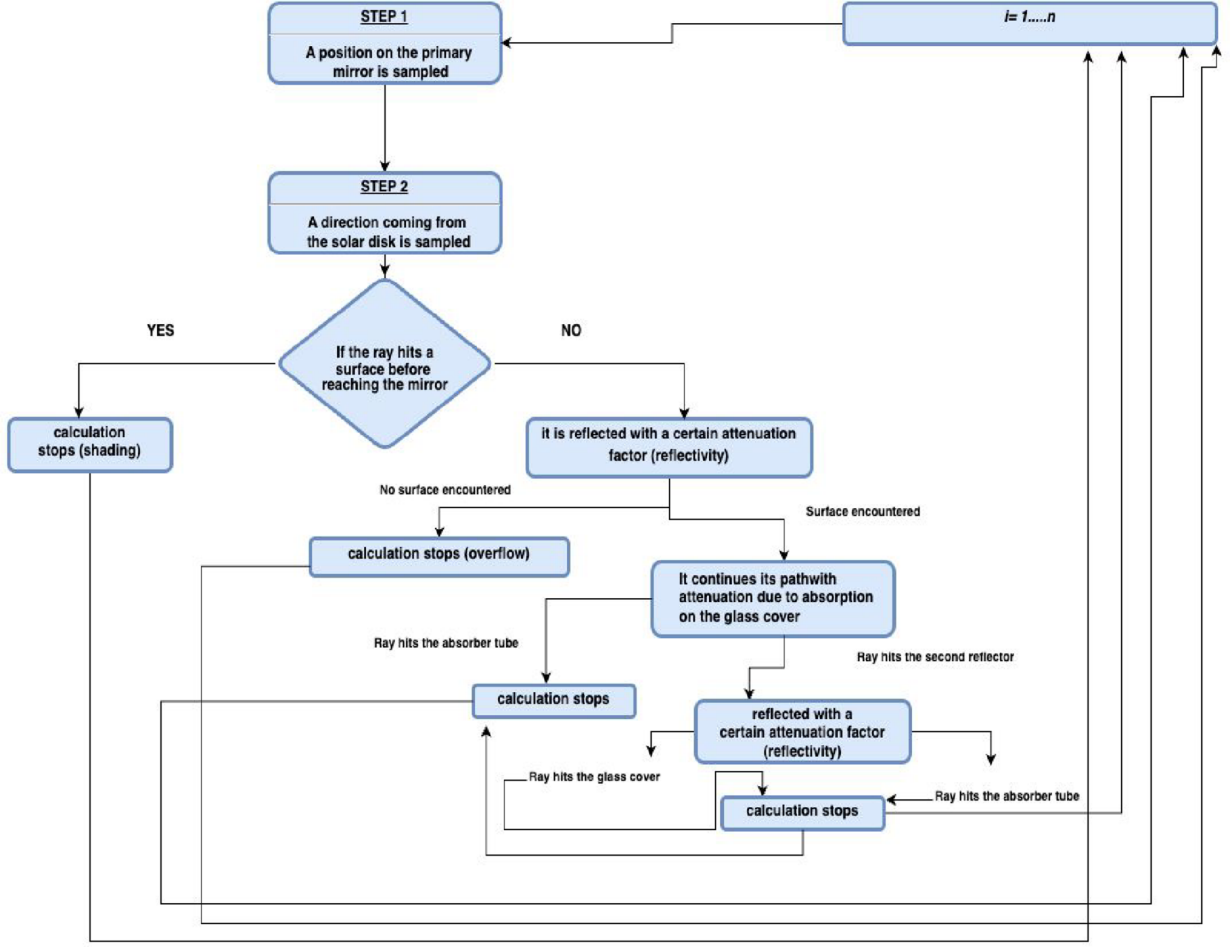
Flowchart of the Monte Carlo ray-tracing algorithm implemented in OPSOL code.

The integral (1) can be written using the Monte Carlo method in a recursive form by applying the importance sampling technique; the general algorithm is summarized in Fig. 5.

After each iteration *i*, a new ray is randomly sampled and consequently the different quantities are re-evaluated and updated until the maximum number of random samplings N is reached. The convergence of the results becomes more accurate as N increases, since a larger number of samples ensures better statistical reliability.

**Fig. 6:**
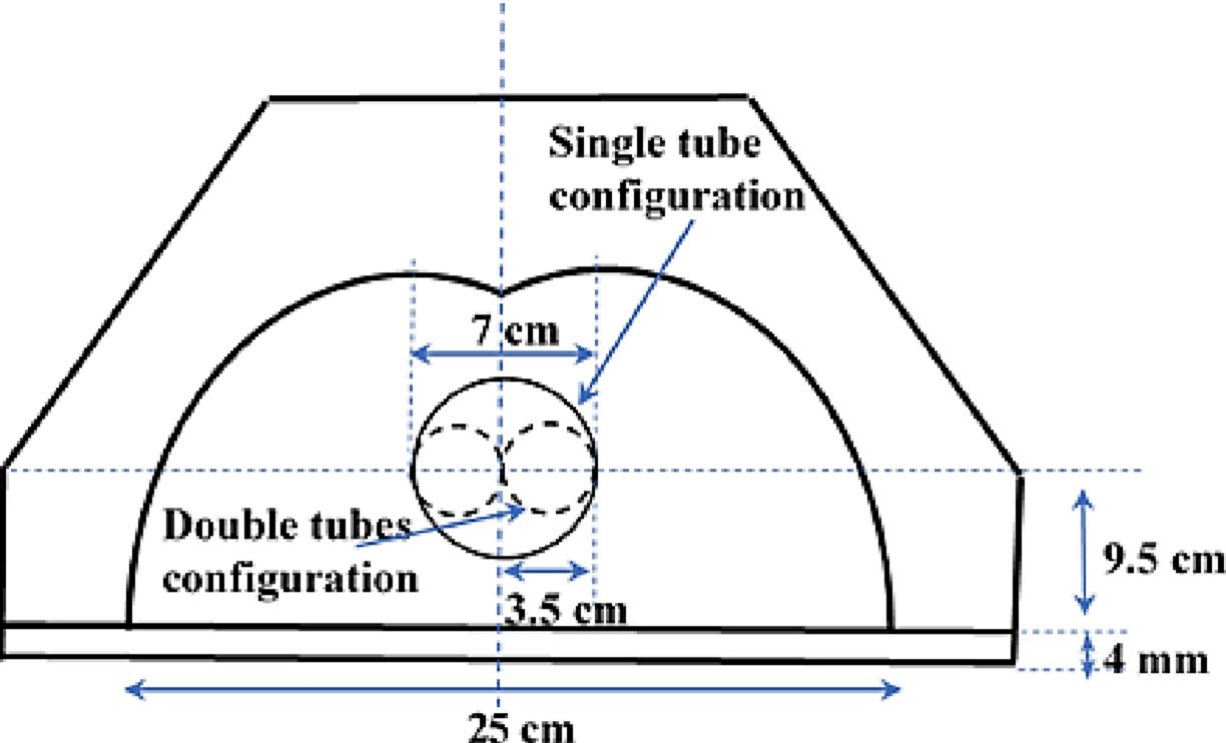
Geometric configuration of the receiver.

The geometric configuration of the receiver is illustrated in Fig. 6. For the single-tube design, we used an absorber tube with a standard diameter of 7 cm. For the dual-tube design, each tube was sized so that its combined lateral surface area was equivalent to that of a single tube, resulting in a diameter of 3.5 cm per tube.

**Fig. 7:**
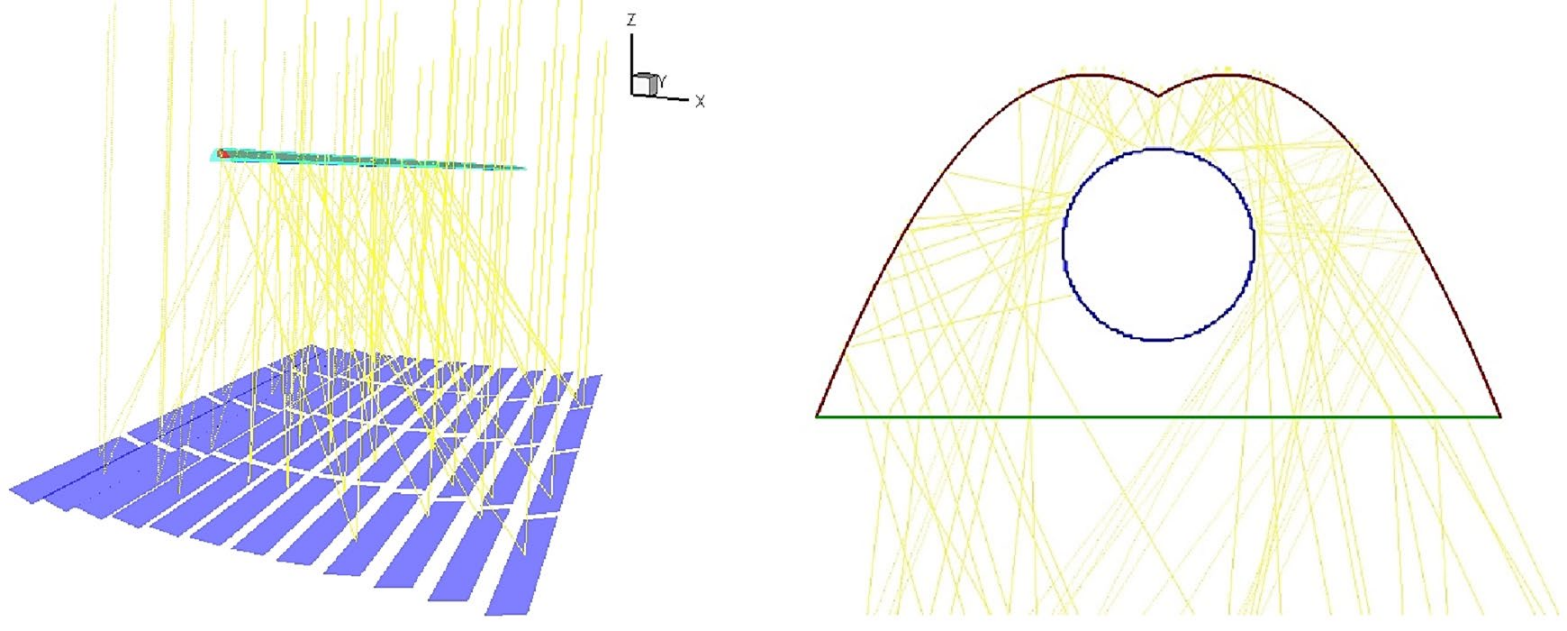
Simulation of the optical interactions between sunrays and different LFR optical components using the OPSOL code.

The MCRT algorithm can be employed to compute various quantities, mainly for evaluating the optical efficiency, rays lost due to overflow or shading, as well as the solar flux absorbed by the glass cover and the secondary reflector after each interaction with them, respectively. In OPSOL code, the same algorithm can also be used to assess the sensitivities to geometric parameters, errors due to irregularities in the reflective surface, and pointing errors. The concentrator’s optical efficiency represents the ratio of the solar energy absorbed by the absorber tube to the energy received at the aperture of the primary mirrors (DNI). Figure 7 shows the optical interactions between sunrays and different LFR optical system using the OPSOL code.

### Thermal modelling (CFD-based approach)

Within the receiver components, the heat transfer occurs by conduction, convection and radiation. The heat transfer model describing the coupled heat and mass transfer is based on the classical laws of mass, momentum, and energy conservation. Within the air cavity, a Newtonian fluid with constant physical properties is described using the incompressible Navier–Stokes equations, incorporating the Boussinesq approximation to represent thermal density variations. Regarding the symmetry along the receiver length, the 2D simulation model can be sufficient to describe the detailed heat transfer inside the air cavity (mainly by convection and radiation). The main specific assumptions on which our numerical model is based can be summarized as follows:

- Since the thermal losses occur mainly from the lateral surface of the absorber tube, the circulation of the heat transfer fluid inside the absorber tube is not considered; consequently, the absorber tube is treated as a fixed-temperature wall.
- The receiver is assumed to be sufficiently long with no end effect, so that axial heat and mass transfer can be neglected (2D approximation).
- All surfaces are assumed to be gray, and the S2S (surface-to-surface) radiation model is used to simulate the radiative heat exchanges within the cavity.
- The fraction of the solar flux absorbed by the glass cover, the secondary reflector, and the absorber tube is treated as a volumetric heat source.

It should be noted that some assumptions represent an idealization of reality. Consequently, they may introduce uncertainties that could quantitatively affect the accuracy of the results. However, the comparisons between the two configurations remain valid, as the simulations are carried out under identical conditions.

- Equation of mass conservation:

2 $$\:\frac{\partial\:\rho\:u}{\partial\:x}+\frac{\partial\:\rho\:v}{\partial\:y}=0$$

- Equations of momentum (Navier-Stokes) :

3 $$\:\frac{\partial\:\rho\:{u}^{2}}{\partial\:x}+\frac{\partial\:\rho\:uv}{\partial\:y}=-\frac{\partial\:p}{\partial\:x}+\frac{\partial\:}{\partial\:x}\left(\mu\:\frac{\partial\:u}{\partial\:x}\right)+\frac{\partial\:}{\partial\:y}\left(\mu\:\frac{\partial\:u}{\partial\:y}\right)\:\:\:\:$$

4 $$\:\frac{\partial\:\rho\:uv}{\partial\:x}+\frac{\partial\:\rho\:{v}^{2}}{\partial\:y}=-\frac{\partial\:p}{\partial\:y}+\frac{\partial\:}{\partial\:x}\left(\mu\:\frac{\partial\:v}{\partial\:x}\right)+\frac{\partial\:}{\partial\:y}\left(\mu\:\frac{\partial\:v}{\partial\:y}\right)-g\left(\rho\:\left(T\right)-{\rho\:}_{0}\right)$$

- Equation of energy conservation in the air cavity:

5 $$\:u\frac{\partial\:\left(\rho\:{c}_{p}T\right)}{\partial\:x}+v\frac{\partial\:\left(\rho\:{c}_{p}T\right)}{\partial\:y}=\frac{\partial\:}{\partial\:x}\left(\lambda\:\frac{\partial\:T}{\partial\:x}\right)+\frac{\partial\:}{\partial\:y}\left(\lambda\:\frac{\partial\:T}{\partial\:y}\right)+Q$$

- Equation governing energy conservation in the solid components.

6 $$\:\frac{\partial\:}{\partial\:x}\left(\lambda\:\frac{\partial\:T}{\partial\:x}\right)+\frac{\partial\:}{\partial\:y}\left(\lambda\:\frac{\partial\:T}{\partial\:y}\right)+Q=0$$

where *u*, *v*, are the velocity components, p the pressure, *T* the temperature, $\:\rho\:$ the air density, $\:\lambda\:$ the thermal conductivity, and *Q* the heat source. A finite volume approach is employed to numerically solve the governing equations. The domain is partitioned into a grid of control volumes. The model takes into consideration the radiative heat transfer carried out using the Surface-to-Surface approach.

**Fig. 8:**
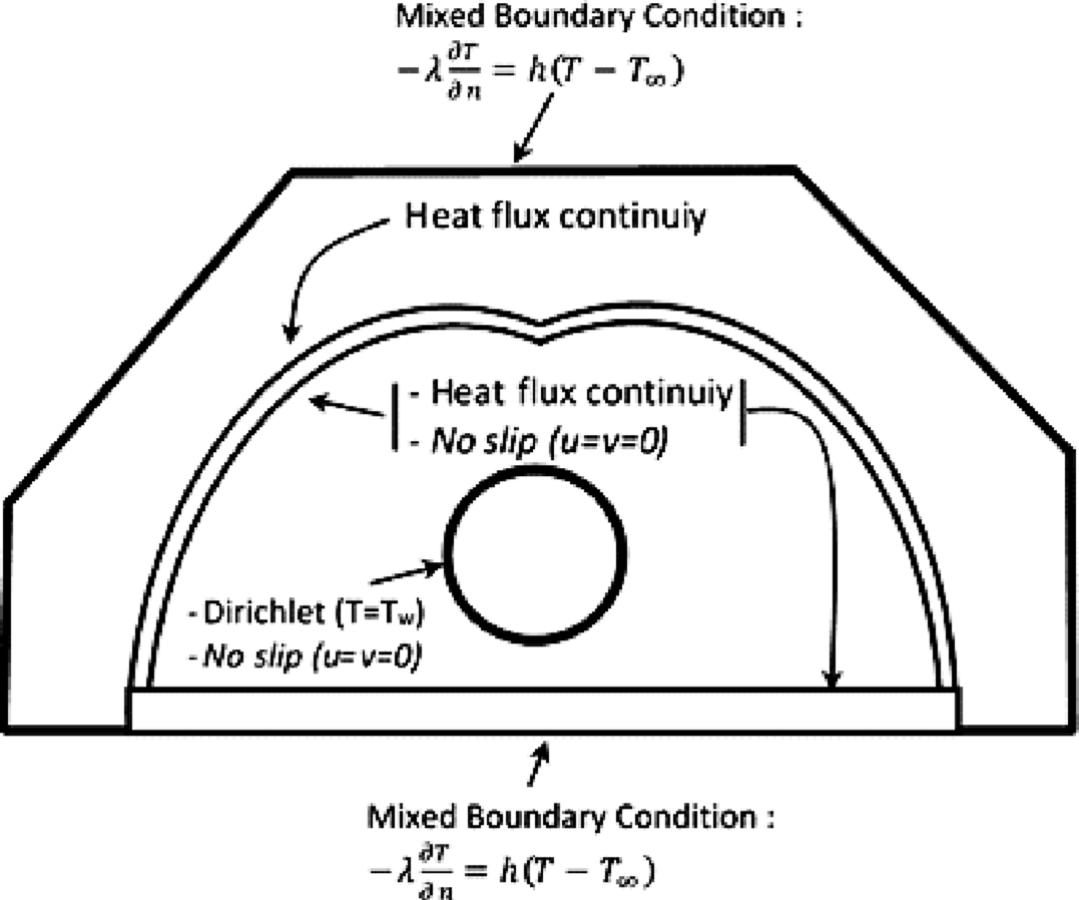
Schematic representation of the boundary conditions used in the numerical model.

The system of equations presented from (2) to (5) constitutes a general formulation expressing the classical conservation laws of mass and energy used in the CFD approach. In particular, Eqs. (5) and (6) represent the principle of energy conservation in the fluid and solid media, respectively, explicitly accounting for both convective and conductive heat transfer mechanisms. Figures (8) shows the schematic representation of the boundary conditions used.

However, certain specific considerations must be taken into account, since in ANSYS Fluent, this equation can describe:

- Either purely conductive heat transfer within solid elements such as the secondary reflector, the glass cover and rock wool. Then, only Eq. (6) is solved with corresponding boundary conditions, assuming pure conduction.
- For the air in the cavity, Eq. (2) to (5) are solved simultaneously, taking into account the boundary conditions.

The radiative heat transfer is modeled using the S2S (Surface-to-Surface) model, where the radiative exchange is computed separately and then incorporated implicitly through the source term Q in Eq. (6). In the case of the air inside the cavity, which is considered a non-participating medium with respect to radiation, the source term Q is assumed to be zero.

Conversely, for the solid components such as the absorber tube, the glass cover, and the secondary reflector, the source term Q must account for both: the radiative fluxes obtained from the radiation exchange model, and the absorbed heat fluxes resulting from solar irradiance coming from the primary mirrors.

The Finite Volume Method (FVM) was used to discretize the governing partial differential equations. The Semi-Implicit Method for Pressure Linked Equations (SIMPLE) algorithm was used to handle the pressure-velocity coupling. The momentum and energy equations were subjected to a second-order upwind scheme for spatial discretization in order to guarantee accuracy and stability. For pressure interpolation, the pressure staggering option (PRESTO!) was employed. When the scaled residuals for each of the governing equations dropped below a strict threshold of 10⁻⁶, the solution was deemed to have converged, guaranteeing a high level of numerical accuracy.

**Fig. 9:**
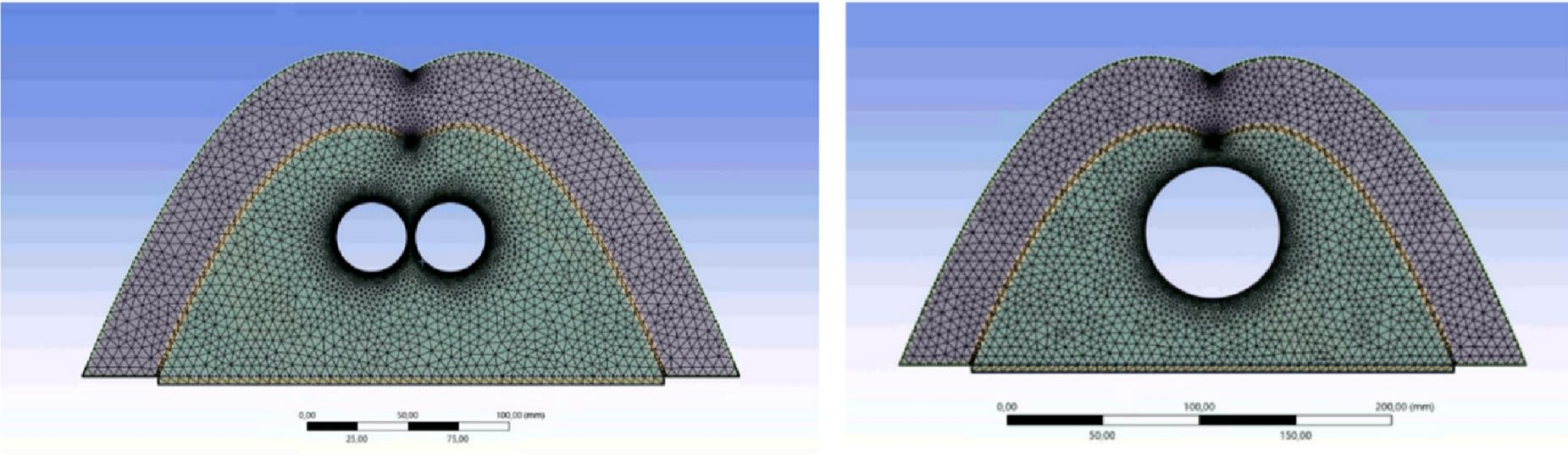
Discretization of the computational domain using triangular mesh.

A two-dimensional computational domain representing the receiver’s cross-section was created and discretized using unstructured triangular elements (Fig. 9). A higher mesh density was applied near geometrical singularities and walls with higher thermal gradient.

To ensure numerical reliability and validate that the results were independent of grid resolution, a mesh independence study was carried out for the conventional single-tube receiver at an absorber temperature of 623 K. Six mesh densities were examined, with node counts ranging from 7904 to 80,274, corresponding to face sizes between 10.0 mm and 1.0 mm.

The global heat flux on the absorber surface was selected as the principal evaluation parameter.

**Fig. 10:**
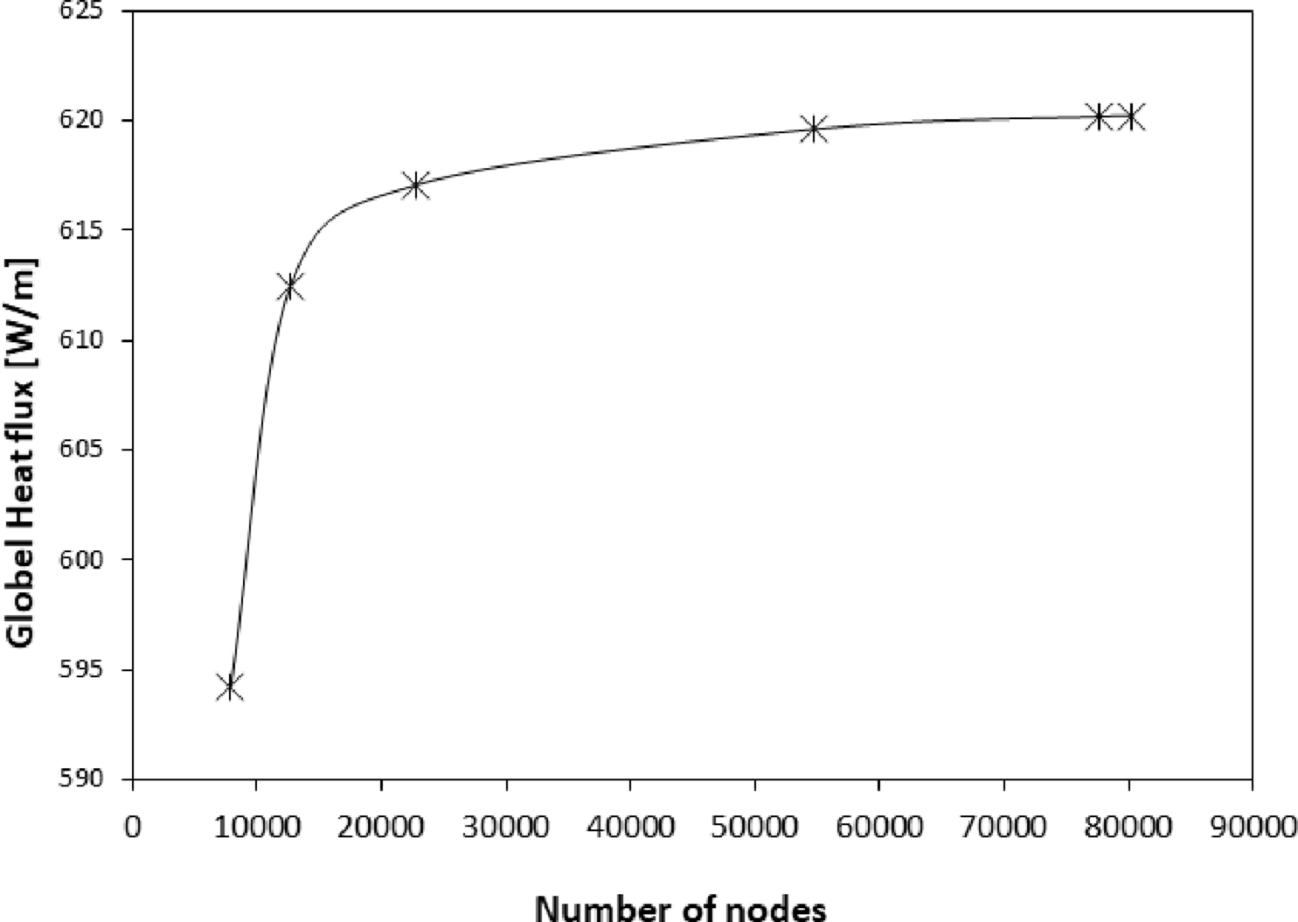
Mesh independence study for the single-tube receiver: variation of the estimated global heat flux on the absorber tube with mesh size.

The estimated global heat flux rises sharply with mesh refinement up to about 54,700 nodes, as Fig. 10 illustrates, after which the values progressively converge. The mesh with about 54,700 nodes (face size ≈ 3 mm) was therefore chosen for all ensuing simulations since it offers the best trade-off between computational expense and numerical precision^23^.

### Experimental setup

**Fig. 11:**
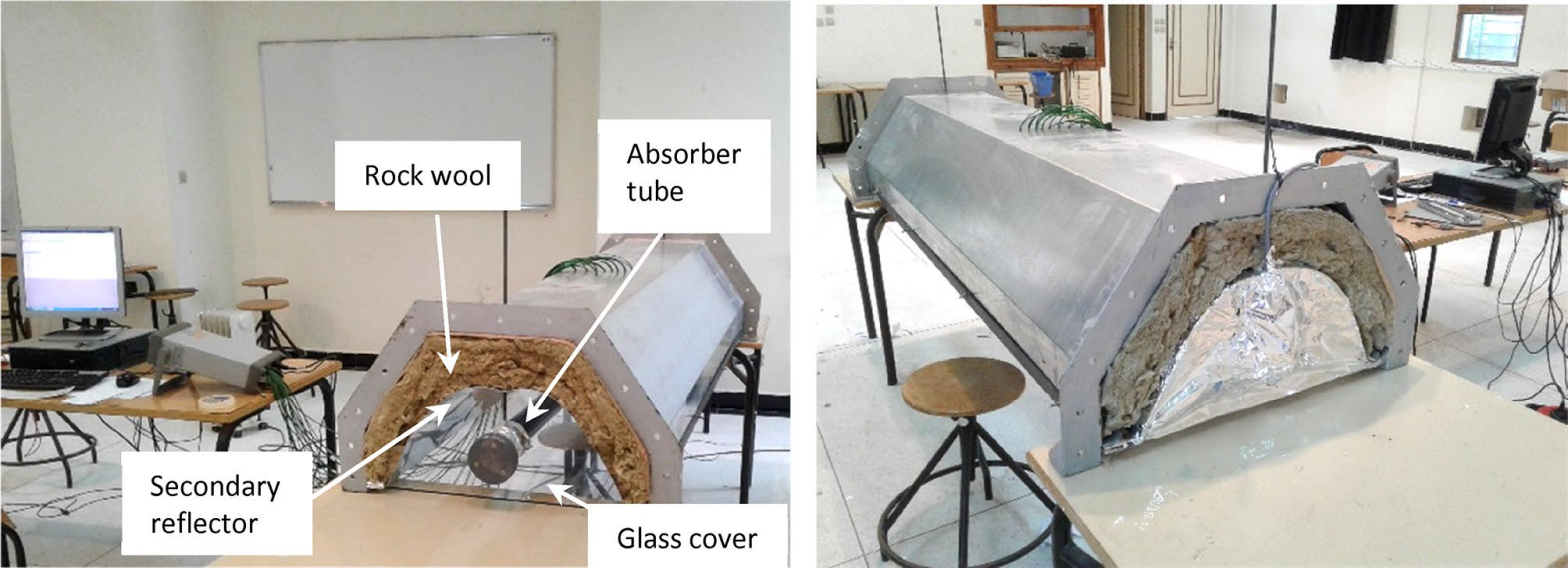
Laboratory-scale single-tube CPC receiver prototype. Modified Ref^19^.

The CFD model was validated by reproducing the experimental conditions of a laboratory-scale single-tube CPC receiver prototype (Fig. 11), previously tested for experimental thermal performance evaluation^19^. The design, inspired by the CHAMS project^20,22^ reproduces the actual transverse dimensions, namely the receiver aperture width, absorber tube diameter, and insulation thickness, while the receiver length was reduced to 2 m for experimental convenience. The receiver is thermally insulated with rock wool and equipped with a 0.5 mm polished aluminium CPC reflector (reflectivity = 0.95). A 4 mm borosilicate glass cover, separated from the metal frame by insulating tapes, limits thermal bridging. The 7 cm steel absorber tube, coated with black nickel, ensures efficient absorption. Both ends of the receiver and cavity are sealed and insulated to minimize end effects, maintaining isothermal conditions along the receiver axis. Table 1 represents the main physical characteristics of the receiver components.

**Table 1:** Main physical characteristics of the receiver components.

Mirrors reflectivity	secondary reflector reflectivity	Glass absorptivity	Glass cover transmissivity	Absorber tube emissivity	Rock-wool conductivity (W/m K)	Glass cover conductivity (W/m K)
0.93	0.95	0.9	0.9	0.21	0.04	1.2

**Fig. 12:**
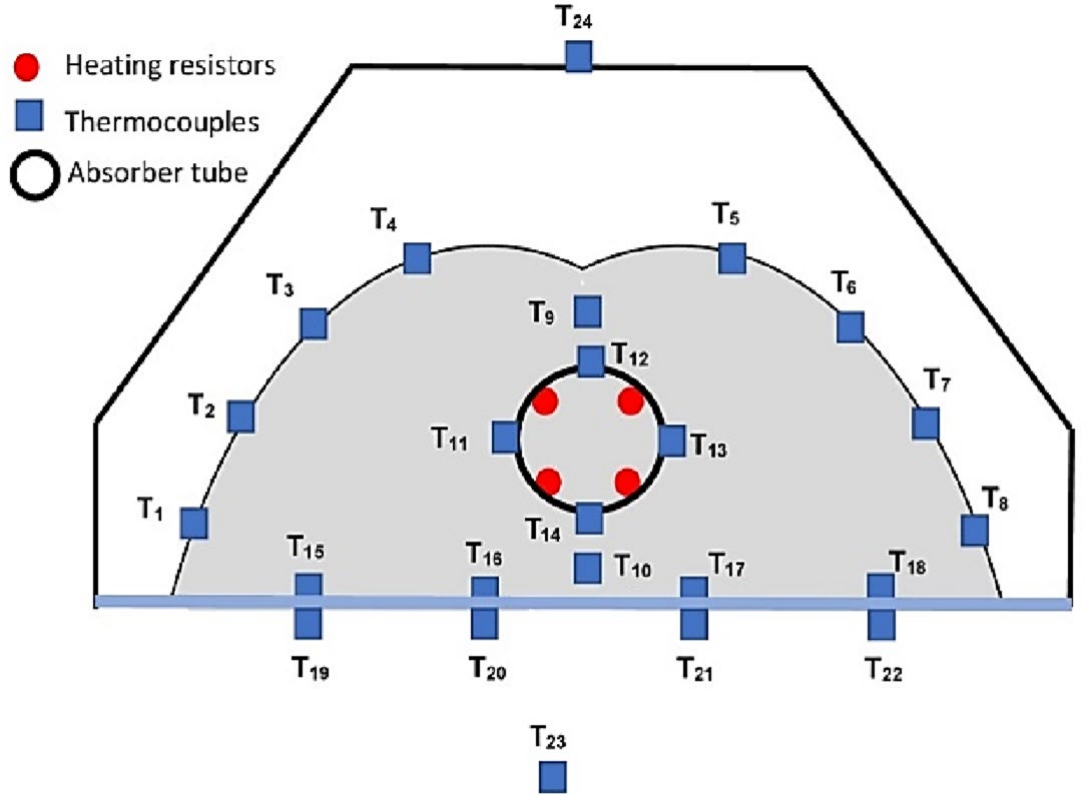
Layout of thermocouples and heating resistors inside the experimental device. Modified Ref^19^.

The receiver’s temperature was measured using 24 high-precision K-type thermocouples distributed over various components (Fig. 12): 8 on the secondary reflector, four on the absorber tube’s lateral surface, 8 on the inner and outer sides of the glass cover, and two inside the air cavity. Additional thermocouples measured the ambient temperature and the external receiver cover temperature. The K-type Chromel-Alumel thermocouples used (0.2 mm junction diameter) provide accurate readings from 250 °C to 1372 °C with an error between 0.75% and 2.2%. Thermal paste and adhesive tape were used to ensure good contact and minimize resistance at the measurement points.

Heating is simulated using 4 electrical resistors of 1 kW inserted into the absorber tube, eliminating the need for a circulating heat transfer fluid. Once thermal equilibrium is reached, the supplied electrical power compensates only for the receiver’s thermal losses. This method allows precise determination of heating power through simple voltage and current measurements. The setup includes a DC power regulation system (Keysight 6675 A, 0–120 V/0–18 A) with high accuracy, and 24 thermocouples connected to an Agilent data acquisition unit for real-time monitoring. Further details can be found in reference^19^.

The electrical energy supplied serves a dual purpose: it increases the receiver’s temperature and compensates for heat losses to the external environment. Over time, the system reaches thermal equilibrium, characterized by stabilized temperatures, indicating that steady-state conditions have been achieved. Therefore, the electrical power input exclusively serves to counteract thermal losses. As part of the experimental investigation, several tests were conducted to validate the CFD thermal model. During each test, a consistent electrical power input was applied to simulate the solar energy absorbed by the absorber tube. For each test, the 24 temperatures were recorded once a steady state was reached.

## Results and Discussion

The optical behaviour of the solar concentrator is simulated for a typical day (June 22, Meknes, Morocco). In the CFD-thermal model, the heat exchange calculation within the heat transfer fluid inside the absorber tube is not required.

### Optical performance insights

**Fig. 13:**
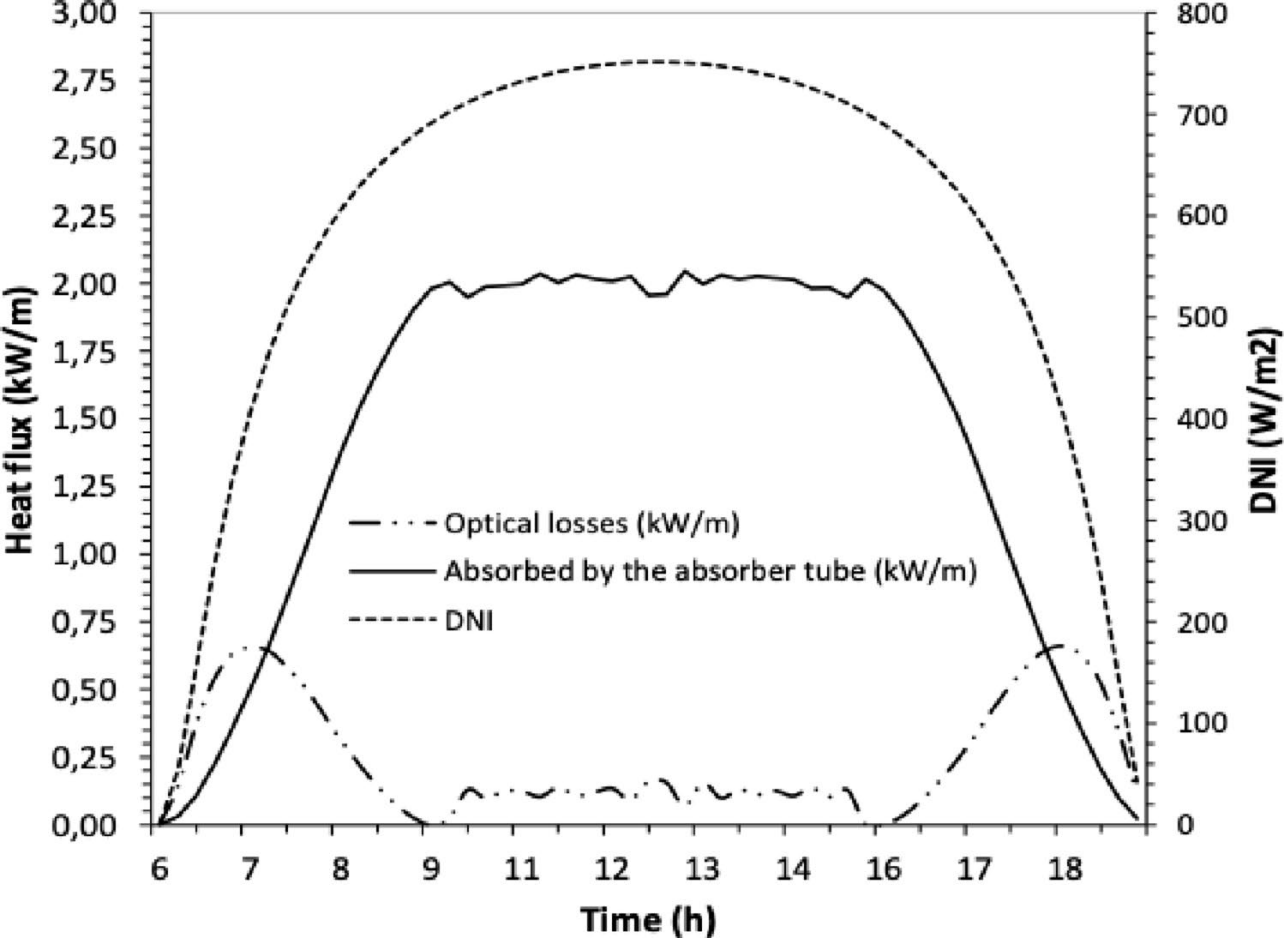
Solar flux absorbed by the receiver tube and optical losses of the whole concentrator.

**Fig. 14:**
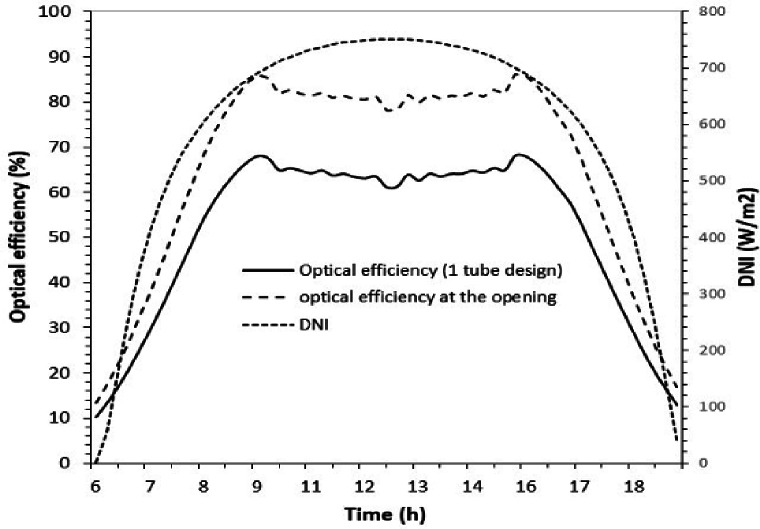
Optical efficiency of the linear Fresnel concentrator over the day.

The results are presented in Fig. 13 (case of single tube design) in terms of solar flux absorbed by the receiver tube, as well as optical losses due to blockage and shading. Figure 14 illustrates the corresponding optical efficiencies for the same simulated day. In both Figs. 13 and 14, we include also the evolution of the direct normal solar radiation (DNI) according to time.

**Fig. 15:**
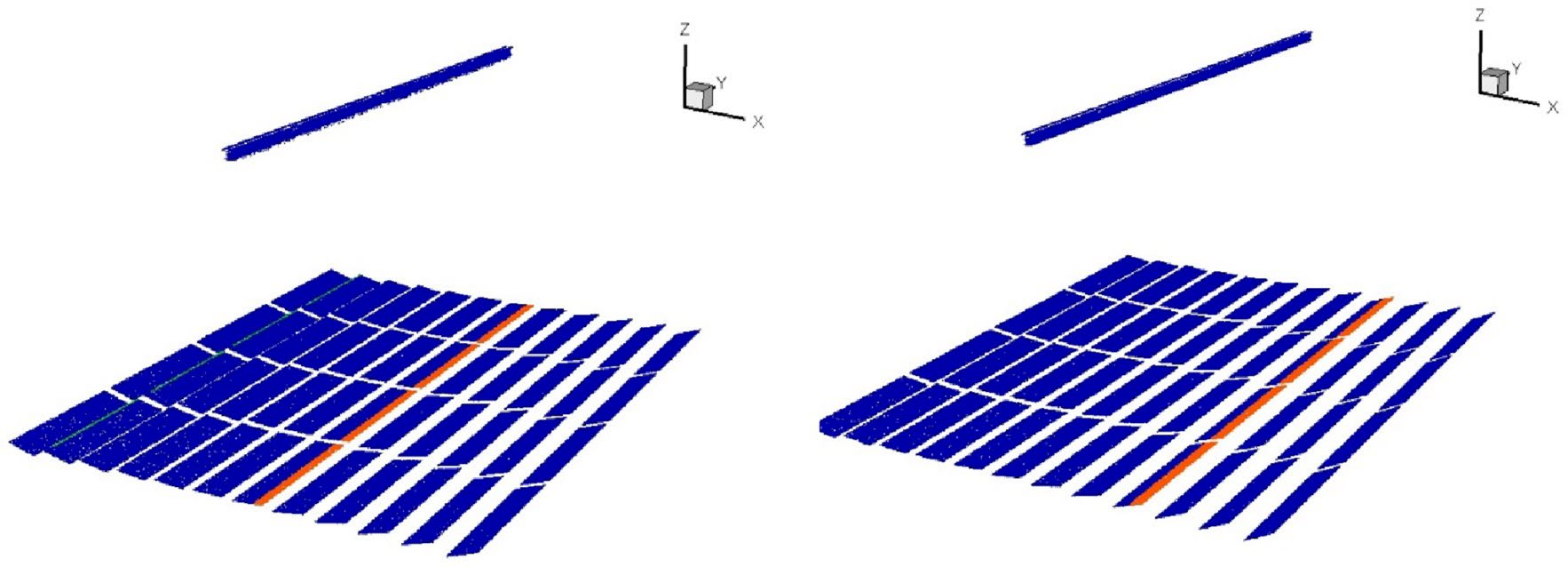
Receiver shadow effect on the surface of the mirrors: at 11 h (right) and 13 h (left).

These results clearly show how the optical performance of this type of concentrator varies throughout the day. The overall optical efficiency of the concentrator remains practically constant, around 65%, for a long period from 9 h to 16 h. The sawtooth-shaped fluctuations are attributed to the periodic passage of the receiver’s shadow over the mirror surfaces and the separating gaps (Fig. 15). These results are particularly important because they demonstrate that, despite the variations in the incident solar power (DNI), the geometric configuration of the concentrator guarantees the stability of the thermal power received by the absorber tube, around 2 kW/m² over the period from 9 h to 16 h. As shown in Fig. 9, the decrease in optical performance outside this period is due to optical losses caused by blocking, shading, and cosine effects, mainly at low solar radiation incidence angles.

**Fig. 16:**
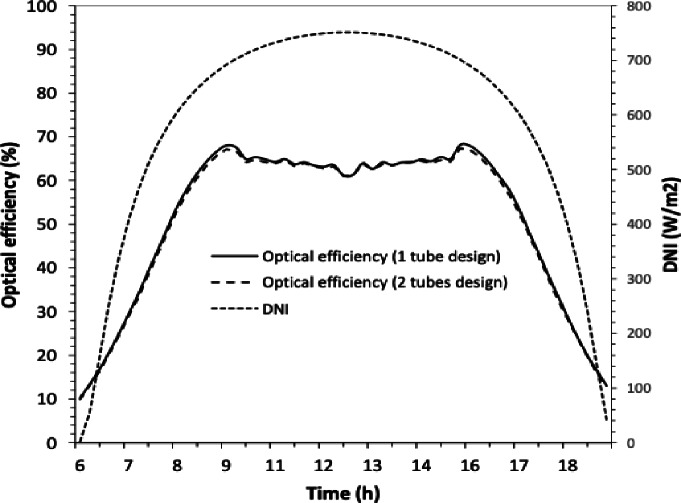
Overall optical efficiency comparison between single-tube and dual-tube designs.

For the dual tube design, Fig. 16 shows very similar optical behaviour both quantitatively and qualitatively. This is explained by the optimal design of the CPC-shaped secondary reflector^22^, which is configured in such a way that, after multiple reflections, all solar rays eventually reach the absorber tube. Consequently, when both tubes are placed in the same position within the cavity, they intercept almost the same amount of solar radiation.

**Fig. 17:**
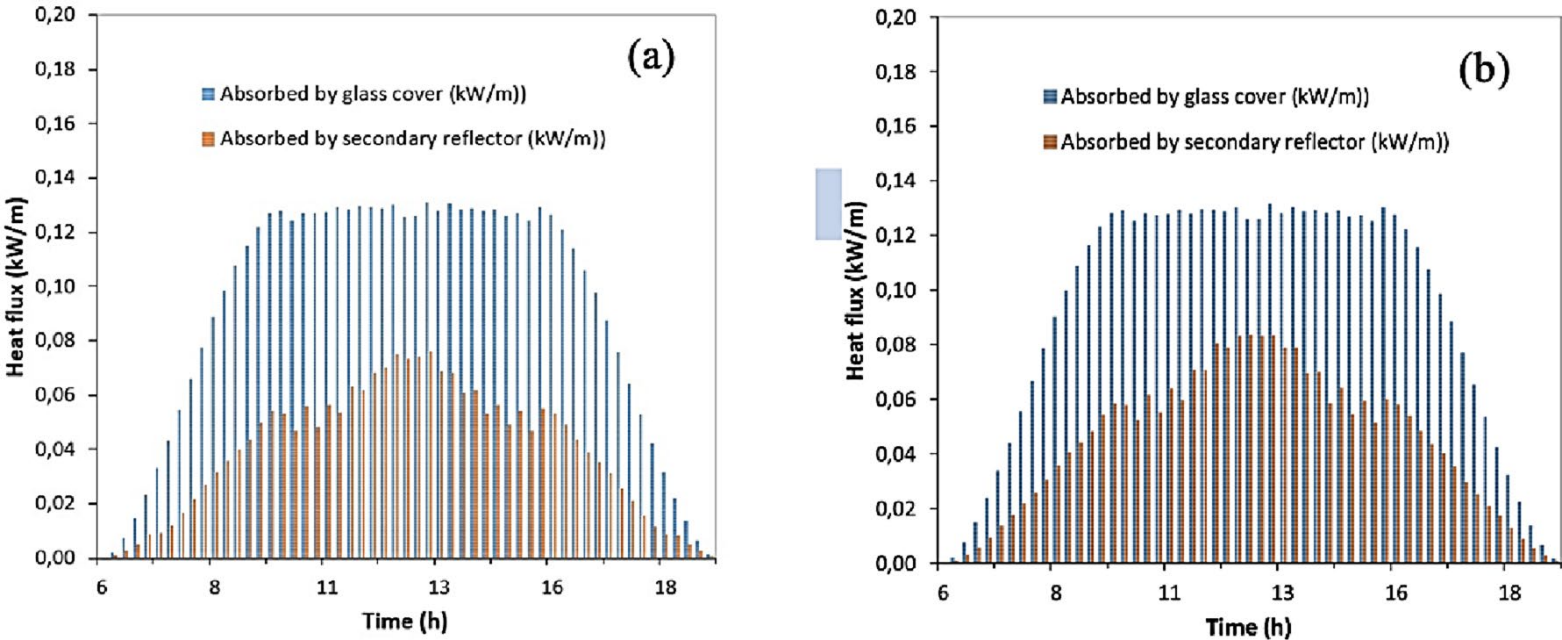
Heat flux absorbed by the glass cover and secondary reflector (a) single tube design,(b) dual tube design.

Figure 17 illustrates, respectively for both designs, the impact of solar radiation absorption properties of the glass cover and the secondary reflector on the overall optical efficiency of the concentrator. Since these components are not perfect reflectors, a portion of the incident solar radiation is absorbed before reaching the tube. From an optical viewpoint, this absorption acts as an attenuation factor, reducing the overall optical efficiency of the concentrator compared to the optical efficiency at the receiver aperture.

### **Thermal behaviour insight**

**Fig. 18:**
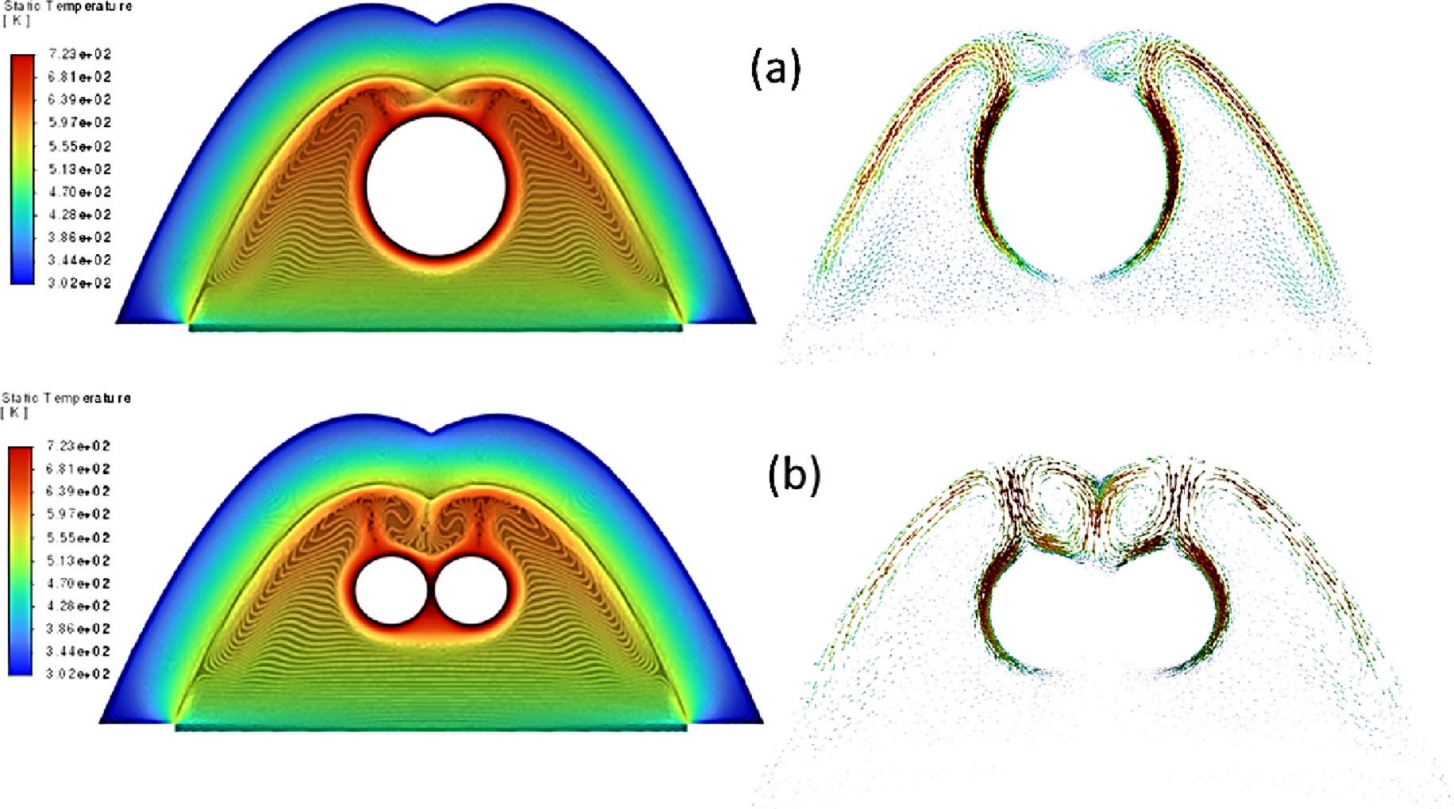
Temperature distribution and velocity within the receiver cavity for the (a) conventional single-tube, (dual-tube).

The CFD results revealed a significant temperature gradient within the receiver cavity. Figure 18 shows the 2D temperature and velocity distribution, highlighting the formation of convective currents. As the air near the absorber tube heats up, it rises, transferring a part of its heat through the secondary reflector. It then descends, completing its cooling process by dissipating heat through the glass cover. Additionally, a pronounced thermal stratification is observed, with the hottest area concentrated at the top of the cavity and the coolest area near the glass cover at the bottom. This natural stratification plays a key role in reducing thermal losses in this configuration compared to conventional flat-plate solar collectors.

**Fig. 19:**
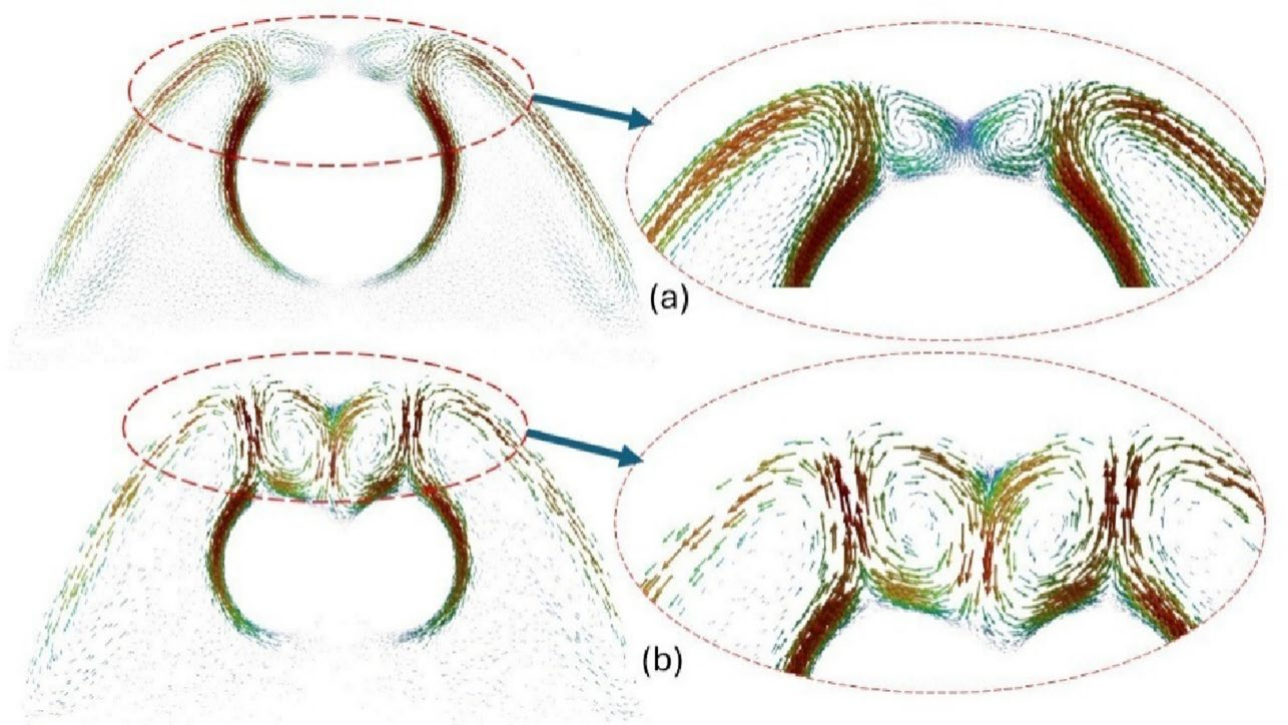
Detailed velocity field illustrating the formation of symmetrical vortices: (a) single-tube configuration, (b) dual-tube configuration.

At the upper part of the absorber tube, two symmetrical vortices form, mainly due to the strong temperature gradients between the tube and the secondary reflector (Fig. 19). These gradients induce localized convective movements in the fluid, generating stable vortices. The formation of these vortices is further enhanced by the geometric singularity of the secondary reflector at the top of the tube, which alters the flow profile and promotes the appearance of recirculation zones. This phenomenon directly affects local heat transfer by enhancing thermal mixing in the upper part of the cavity.

### Thermal model validation results

**Fig. 20:**
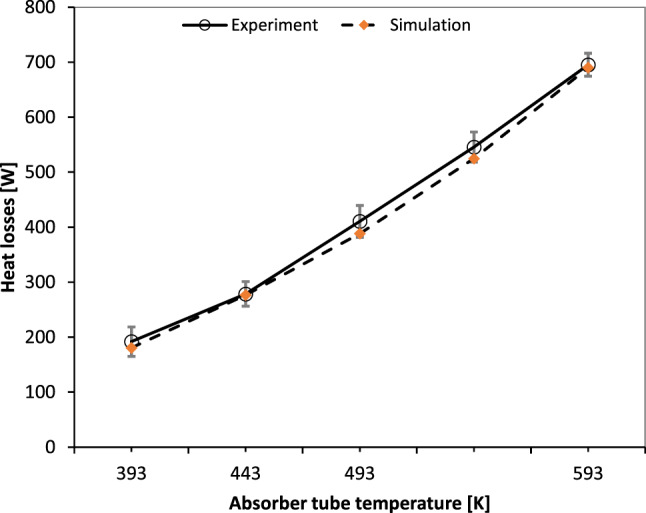
Comparison between experimental measurements and numerical results from the CFD model for total heat losses (W), for a single tube configuration. Error bars indicate the combined uncertainty due to temperature measurements and the input electrical heating power used during the experiments.

To compare the experimental results with the CFD simulations, the CFD model was configured to reproduce the same conditions as those of the experiment. Figure 20 illustrates a comparison results, focusing on overall heat losses. Error bars indicate the combined uncertainty due to temperature measurement and the input electrical heating power used during the experiments. When ignoring the combined experimental measurement uncertainty, the average relative deviation between the measured thermal losses and the model predictions remains below 5%. This excellent agreement demonstrates that the proposed method reliably reproduces the global heat balance with high accuracy. Consequently, the model represents a robust and efficient alternative to direct experimental measurements. Even when the experimental uncertainty is taken into account, the deviation increases to a maximum of only 9%, further confirming that the CFD approach provides a robust predictive capability within an acceptable margin of error.

### Single-Tube versus Dual-Tube performance

**Fig. 21:**
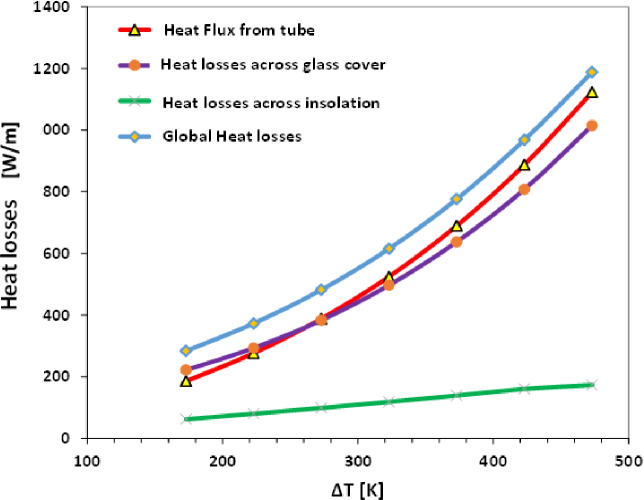
Heat flux and heat losses within the receiver cavity for single-tube design.

We conducted a series of simulations to evaluate the thermal performance of the single and dual tube receivers according to the absorber tube temperature ranging between 473 K and 773 K. In each simulation, we set the temperature of the absorber tube as a boundary condition in the CFD model. To take into consideration the fraction of solar radiation absorbed by the glass and secondary reflector, we relied on the optical simulation results presented above. The considered heat flux values correspond to those recorded between 9 h and 16 h, namely 0.13 kW/m² for the glass and 0.054 kW/m² for the secondary reflector. This corresponds to a total thermal power received by the absorber tube of 2 kW over the same period of the day for both designs, as shown in Fig. 21.

**Fig. 22:**
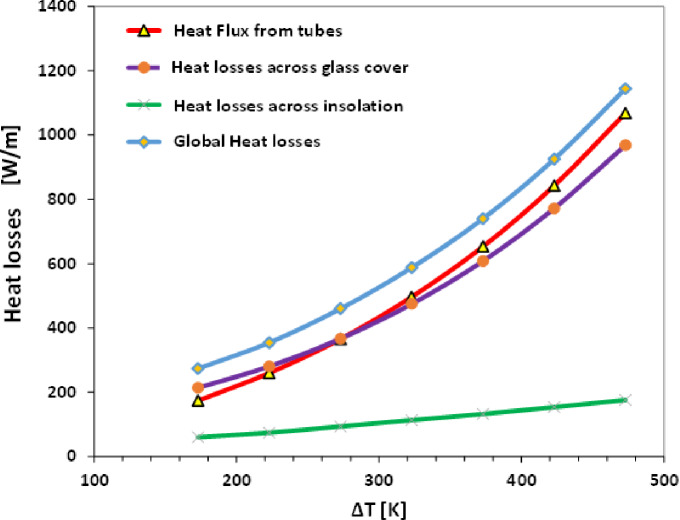
Heat flux and heat losses within the receiver cavity for dual-tube design.

For both design, Figs. 21 and 22 illustrate different heat fluxes obtained per linear meter of the receiver, including the heat flux emitted by the absorber tube, heat losses through the glass, across the upper part of the receiver, and the global heat losses toward the external medium. These results are derived considering the three modes of heat transfer: convection, conduction, and radiation. The global heat losses obtained are ranged, depending on the absorber tube temperature, between 284 W/m and 1188 W/m for single tube design, and 273 W/m to 1144 W/m for dual tube design.

**Fig. 23:**
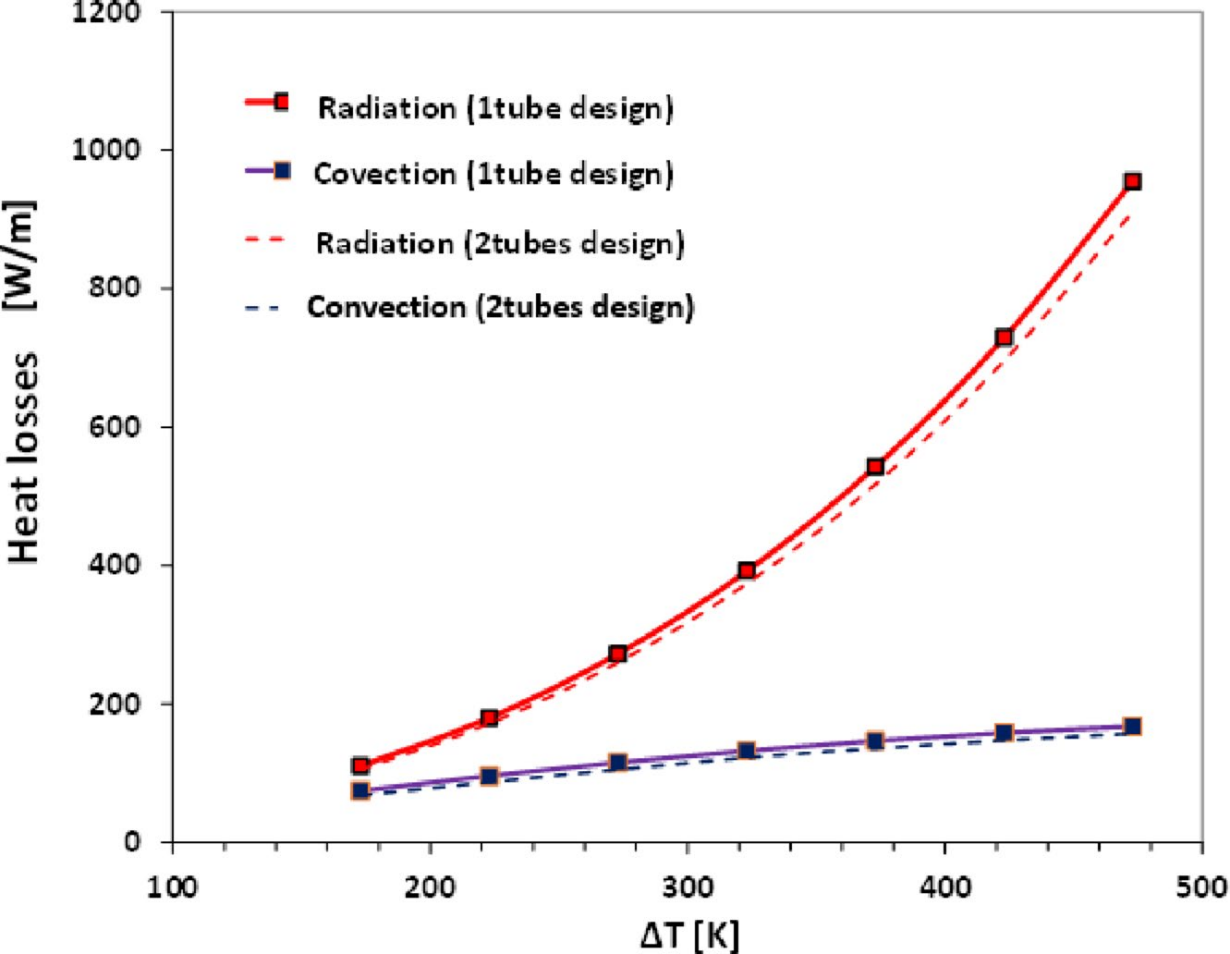
Comparison between the heat losses by convection mode and radiation mode within the receiver cavity.

Nevertheless, it is crucial to point out that the solar heat flux that the glass cover and secondary reflector absorb contributes significantly to the overall heat balance and, consequently, in the total heat flux emitted by the receiver. Indeed, as depicted in Figs. 21 and 22, in addition to the heat flux emitted by the tube, the global heat losses to the external medium also include the fraction of energy re-emitted by the glass and the secondary reflector. This explains why total heat losses exceed the heat flux emitted exclusively from the outer tube surface. For the same reason, when the absorber tube temperature is below 523 K, heat losses from the glass exceed those from the absorber tube. This finding is particularly noteworthy, as it highlights that although the optical absorption properties of the glass and secondary reflector reduce the receiver’s optical efficiency; they can still be considered beneficial for its overall thermal performance. Thus, a compromise can be found to optimize both optical and thermal behaviour.

Figure 23 demonstrates that, for the range of temperature difference considered here, the primary mode of heat transfer within the cavity occurs predominantly by radiation, which accounts for more than 60% of total losses when the temperature difference reaches 173 K. This contribution increases to over 80% for temperature differences exceeding 400 K. On the other hand, for both designs, heat losses, including radiation and convection, occur mainly through the glass cover, which represents 80% of the total losses.

**Fig. 24:**
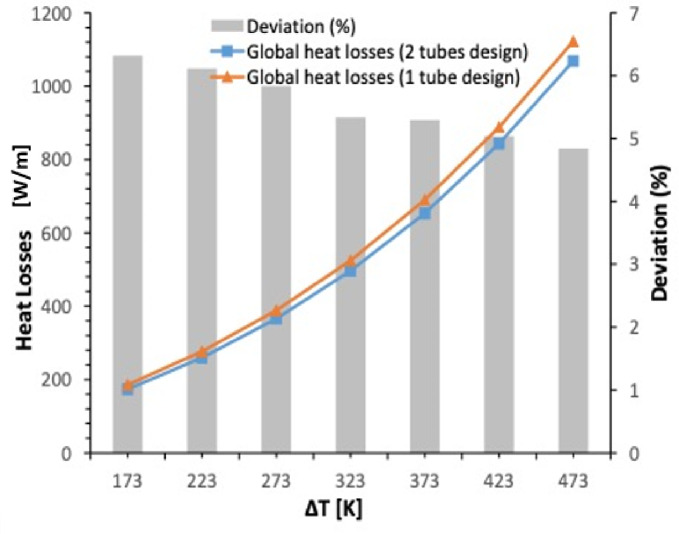
Comparison of global heat losses between the dual-tube and single-tube designs.

**Fig. 25:**
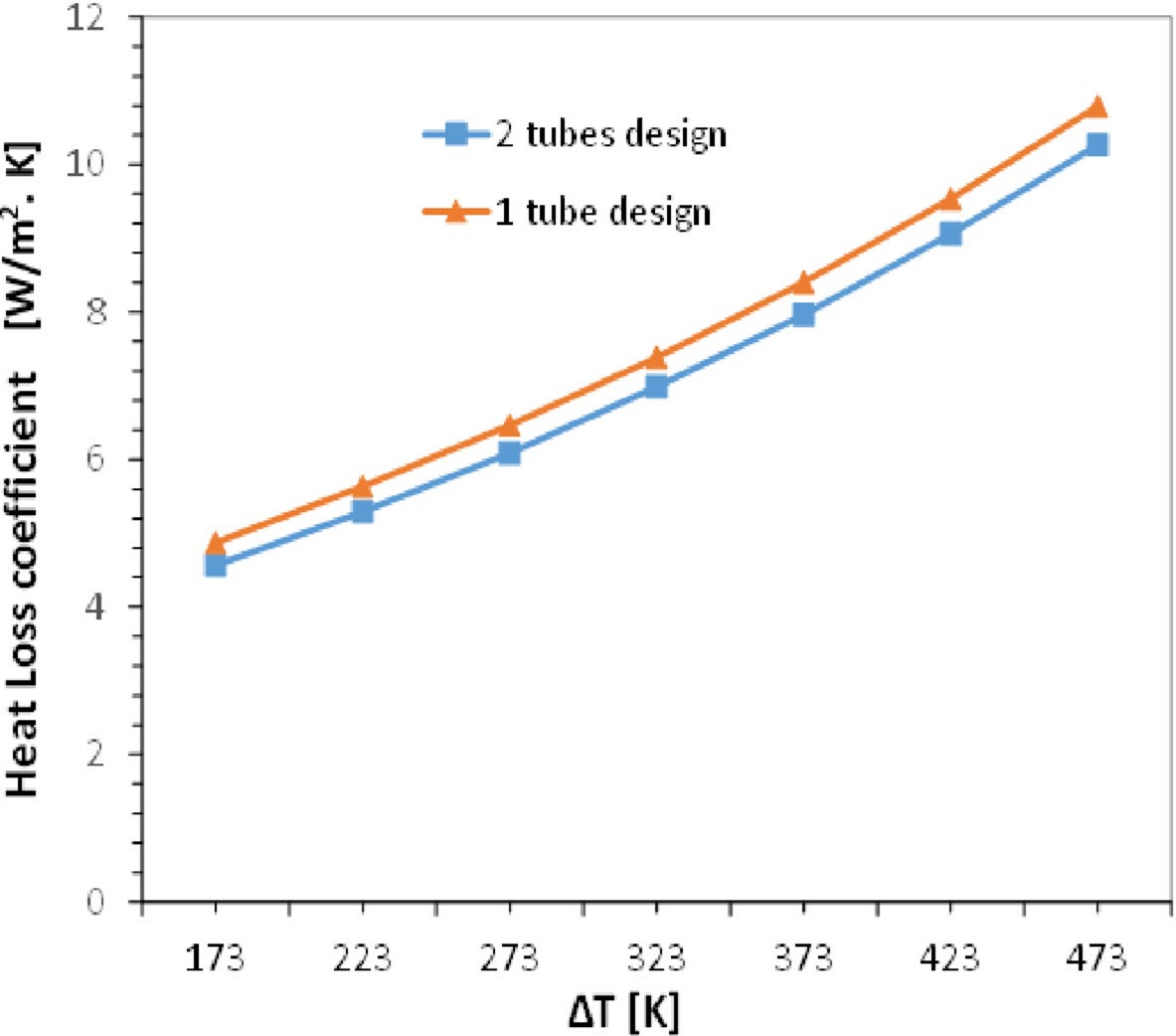
Overall heat loss coefficient for dual-tube and single-tube designs.

The comparison between the two receiver designs shown in Fig. 24 indicates that, under the investigated operating conditions and modelling assumptions, the double-tube configuration yields slightly lower thermal losses than the single-tube configuration. The numerical results suggest a reduction in the overall thermal losses of about 5% compared with the single-tube design. However, with reference to Fig. 20, such differences may fall within the range of uncertainties associated with the modeling assumptions, numerical approximations, and calculation procedures inherent to the CFD approach. Therefore, the reported results should be interpreted with appropriate caution.

This trend is nevertheless physically consistent with the geometrical characteristics of the double-tube configuration. For the same cavity geometry, aperture, and CPC arrangement, the use of two smaller-diameter tubes allows the lower surface of the absorber tubes to be positioned farther from the glass cover. This increased spacing may contribute to reducing radiative and convective heat exchange between the absorber tubes and the glass cover, thereby limiting heat transfer to the ambient.

The corresponding overall heat loss coefficient, relative to the absorber tube surface area, is presented in Fig. 25. It is predicted to range from 4.6 to 10.3 W/m²·K for the double-tube configuration, compared with 4.9 to 10.8 W/m²·K for the single-tube configuration. These values indicate a slight thermal advantage for the double-tube design within the adopted numerical framework. Nevertheless, since the difference between the two configurations is relatively small, the comparison should be interpreted with caution and considered dependent on the specific assumptions, boundary conditions, and simplifications used in the present CFD model.

## Limitations and recommendations for future research

While the present study provides valuable insights and develops effective numerical tools for analysing receiver performance, several limitations should be acknowledged. First, the current modelling approach assumes a simplified representation of the receiver geometry and does not fully capture spatial variations in solar flux distribution. This assumption may lead to localized discrepancies in predicting heat transfer rates and thermal losses. In addition, the comparative thermal results obtained for the single- and dual-tube configurations should be supported by a formal uncertainty quantification in order to confirm the advantage of the double-tube configuration.

To address these limitations, future research should focus on optimizing the receiver geometry using the developed numerical tools. An important enhancement to the present approach would involve explicitly accounting for the spatial non-uniformity of solar flux over the receiver surfaces, enabling more accurate predictions of local heat transfer and improving the overall design efficiency. Accounting for this factor would also enable evaluation of temperature-induced inhomogeneity effects on thermo-mechanical stresses in the glass cover, thereby contributing to the prevention of failure or cracking. It is important also to note that, the study mainly focuses on thermal aspects, without incorporating detailed economic or operational cost analyses.

## Conclusion

Through the present work, we introduced a hybrid numerical–experimental approach for accurately evaluating the overall performance of non-vacuum LFR with a CPC and a transparent glass cover. The originality of this methodology lies in the combined use of MCRT optical model and a CFD thermal analysis, enabling high fidelity simulation of both optical and thermal phenomena occurring with the receiver. This hybrid framework was applied to two receiver configurations: one with a single absorber tube and another with dual absorber tubes.

It is important to note that the experimental validation stage was performed solely for the single-tube configuration, which served as the reference case for model verification. The great agreement between the experimental measurements and the CFD simulations showing deviations below 5% in global heat losses demonstrates the reliability of the model and its ability to accurately reproduce the real thermal behaviour of the receiver. The experimental assessment of the dual-tube receiver is currently in progress and will be presented in a future publication to further consolidate and extend the findings reported here. The key findings of this study can be drawn as follows:

Regarding thermal performance, the simulations suggest that the dual-tube configuration may provide a modest thermal advantage, on the order of about 5%, relative to the single-tube design under the specific assumptions and boundary conditions considered in this work. This tendency is associated with the greater spacing between the smaller-diameter tubes and the glass cover, which may reduce radiative and convective losses. Accordingly, the numerical results indicate that the dual-tube configuration can improve thermal behaviour without noticeably compromising optical efficiency, although this comparative advantage should be considered with due caution until supported by dedicated uncertainty analysis and direct experimental comparison. for temperature differences between 173 K and 473 K relative to ambient, the predicted total heat losses range from 284 to 1188 W/m² for the single-tube receiver and from 273 to 1144 W/m² for the dual-tube design.

In terms of the overall heat loss coefficient, the dual-tube receiver is predicted to achieve values between 4.6 and 10.3 W/m²·K, compared with 4.9 to 10.8 W/m²·K for the single-tube configuration. These values are comparable to, and in some cases slightly lower than, those reported for previously published non-vacuum LFR designs^15^, which suggests the potential benefit of CPC integration and cavity geometry optimization.

Overall, the present results support the relevance of the dual-tube non-evacuated receiver as a promising design option for improving thermal performance while preserving similar optical efficiency. More broadly, this work provides a validated hybrid modelling methodology that can be used for receiver optimization, material and geometry selection, and secondary optics design in linear Fresnel systems. By combining numerical modelling with experimental validation on the reference configuration, the study contributes a practical framework for the analysis and development of next-generation LFR receivers.

## Data Availability

“Data is provided within the manuscript “.

## Abbreviations

MCRT: Monte Carlo Ray-Tracing
CFD: Computational Fluid Dynamics
CSP: Concentrated Solar Power
LFC: Linear Fresnel Concentrator
PTC: Parabolic Trough Concentrator
EOR: Enhanced Oil Recovery
DNI: Direct Normal Irradiance
CPC: Compound Parabolic Concentrator
CEC: Compound Elliptic Concentrator
LFR: Linear Fresnel Reflector

## Symbols

*Q*: Volumetric Heat Source or heat transfer rate, W or W/m^3^
*T*: Temperature, K or °C
*T*_*a*_: Ambient temperature, K
*T*_*s*_: Surface temperature of absorber tube, K
*ρ*: Density of air, kg/m³
*μ*: Dynamic viscosity, Pa·s
*k*: Thermal conductivity, W/m·K
*h*: Convective heat transfer coefficient, W/m²·K
*σ*: Stefan–Boltzmann constant (5.67×10⁻⁸), W/m²·K⁴
*\varepsilon*: Surface emissivity, -
*α*: Absorptivity, –
*τ*: Transmissivity, –
*R*: Reflectivity, –
*p*: Pressure, Pa
*u, v*: Velocity components (x and y directions), m/s
*g*: Gravitational acceleration, m/s²
*θ*: Incidence angle, °
$$\eta_{opt}$$: Optical efficiency, –
$$\eta_{th}$$: Thermal efficiency, –
*U*_*L*_: Overall heat loss coefficient, W/m²·K
